# Invasion and persistence of *Mycoplasma bovis* in embryonic calf turbinate cells

**DOI:** 10.1186/s13567-015-0194-z

**Published:** 2015-05-15

**Authors:** Sibylle Bürki, Véronique Gaschen, Michael H Stoffel, Ana Stojiljkovic, Joachim Frey, Kathrin Kuehni-Boghenbor, Paola Pilo

**Affiliations:** Institute of Veterinary Bacteriology, Vetsuisse Faculty, University of Bern, Bern, Switzerland; Division of Veterinary Anatomy, Vetsuisse Faculty, University of Bern, Bern, Switzerland; Graduate School for Cellular and Biomedical Sciences, University of Bern, Bern, Switzerland

## Abstract

*Mycoplasma bovis* is a wall-less bacterium causing bovine mycoplasmosis, a disease showing a broad range of clinical manifestations in cattle. It leads to enormous economic losses to the beef and dairy industries. Antibiotic treatments are not efficacious and currently no efficient vaccine is available. Moreover, mechanisms of pathogenicity of this bacterium are not clear, as few virulence attributes are known. Microscopic observations of necropsy material suggest the possibility of an intracellular stage of *M. bovis.* We used a combination of a gentamicin protection assay, a variety of chemical treatments to block mycoplasmas entry in eukaryotic cells, and fluorescence and transmission electron microscopy to investigate the intracellular life of *M. bovis* in calf turbinate cells. Our findings indicate that *M. bovis* invades and persists in primary embryonic calf turbinate cells. Moreover, *M. bovis* can multiply within these cells. The intracellular phase of *M. bovis* may represent a protective niche for this pathogen and contribute to its escape from the host’s immune defense as well as avoidance of antimicrobial agents.

**Electronic supplementary material:**

The online version of this article (doi:10.1186/s13567-015-0194-z) contains supplementary material, which is available to authorized users.

## Introduction

The wall-less bacterium *Mycoplasma bovis* is the causative agent of bovine mycoplasmosis, which is responsible for tremendous economic losses in both beef and dairy industries [1]. The clinical spectrum of this disease is broad as it manifests as pneumonia, mastitis, polyarthritis, otitis media and genital disorders [2-5]. Moreover, management of bovine mycoplasmosis is challenging as current vaccines are mostly ineffective [6] and antibiotic treatments generally fail. Furthermore, emergence of *M. bovis* strains resistant to antibiotics, under axenic growth conditions, has been reported [7,8].

Virulence determinants involved in the mechanisms of pathogenicity of *M. bovis* are virtually unknown. Variable surface proteins [9] and the capacity of this bacterium to form biofilms were identified as mechanisms contributing to the persistence of *M. bovis* in its natural environment [10].

*Mycoplasma* spp. are mainly described as extracellular bacteria closely associated with host cells [11,12]. Beyond the well-studied *M. penetrans* [12,13], the ability of several *Mycoplasma* spp. to invade non-phagocytic cells under specific experimental conditions was described [14-20]. Although the role in pathogenicity of the intracellular stage of these bacteria is not yet clear, it deserves to be investigated in more detail to elucidate the molecular mechanisms involved.

The close extracellular association of *M. bovis* with host cells and adhesion characteristics have been described with occasional intracellular localizations in inflammatory cells [21-30]. Studying lung tissues of experimentally infected calves by transmission electron microscopy (TEM), Kleinschmidt et al. recently observed *M. bovis* throughout caseonecrotic foci, in the cytoplasm of degenerating macrophages and the lumina of bronchi but not in the cytoplasm of bronchial epithelial cells [22]. Additionally, van der Merwe et al. observed intracellular *M. bovis* in bovine peripheral blood mononuclear cell populations (PBMC) and red blood cells (RBC) following in vitro infections [31]. Moreover, *M. bovis* antigens were detected inside inflammatory cells, hepatocytes, renal tubular epithelial cells and facial nerve bundles of necropsy tissue samples by immunohistochemistry and by TEM [32]. Consequently, the intracellular stage of *M. bovis* in non-phagocytic cells needs further investigations to strengthen these observations from naturally and experimentally infected animals and cells.

Invasion and persistence of *M. bovis* in phagocytic and non-phagocytic host cells may contribute to the pathogenesis of the bacterium serving as a protection niche evading the host immune response and antibiotic treatment but could also lead to systemic spread within host blood cells. A definitive proof of the ability of *M. bovis* to invade non-phagocytic cells has not been experimentally demonstrated and the development of an in vitro model is essential to dissect the molecular and cellular mechanisms involved in the intracellular survival of *M. bovis* in these cells.

The aim of the present study was to investigate invasion and persistence of *M. bovis* in bovine non-phagocytic cells using an in vitro model. Several complementary approaches including the gentamicin protection assay, considered as the gold standard method for investigating bacterial invasion, chemical blocking of endocytic pathways, fluorescence microscopy, as well as TEM were performed. The results reveal that *M. bovis* is able to invade and persist in bovine turbinate cells. Moreover, *M. bovis* is able to replicate within these cells.

## Materials and methods

### Bacterial strains, primary calf turbinate cells and growth conditions

Strains of *M. bovis* (Table 1) were grown at 37 °C in SP4 medium [33] supplemented with 50 μg/mL cefoxitin sodium salt (Sigma-Aldrich, Buchs, Switzerland) for 24 h in broth medium or for 4 to 5 days on agar plates unless otherwise described. SP4 agar plates were incubated at 37 °C in a humified atmosphere. The *M. bovis* strain JF4278 was selected for microscopy experiments and inhibition assays because it is a field strain isolated from the milk of one of the first cows showing severe mastitis and pneumonia in Switzerland in 2008. The facultative intracellular bacterium *Listeria monocytogenes*, strain JF3263 [34], was used as a control for intracellular growth and was grown on Trypticase Soy Agar supplemented with 5% sheep blood (TSA 5% SB, Becton Dickinson, Allschwil, Switzerland) at 37 °C.

**Table 1:** **Strains of** ***M. bovis*** **used in this study**

**Isolate**	**Origin**		**Year of isolation**	**Reference**
	**Country**	**Source**		
119B96	UK	Lung	1996	[62]
JF4278	Switzerland	Milk	2008	[63]
JF5261	Germany	Milk	2012	This study
L63/93	Switzerland	Lung	1993	This study
01-51020	Canada	Lung	2001	[64]
01-48015	Canada	Lung	2001	[64]

Primary Embryonic Calf Turbinate (PECT) cells were prepared from bovine fetuses. Briefly, cells were collected from 3–5 months old fetuses (crown-rump-length: 25–40 cm). Noses were shortly burned, cut and folded back in order to expose the epithelium. The latter was removed with a sterile curette and cut into small pieces. Subsequently, 0.25% trypsin (1:250) (Biochrom, Berlin, Germany) digestion for 30 min at room temperature with stirring was carried out. Cells were grown and maintained in minimal essential medium (MEM)-Earle medium supplemented with 2.2 g/L NaHCO_3_ (Biochrom, Berlin, Germany) and with 7% fetal calf serum at 37 °C in a 5% CO_2_ atmosphere as previously described [35,36]. The quality of the cells was evaluated by light microscopy and by TEM for characteristic morphological features. Cells were routinely screened to ensure an absence of contamination by mycoplasmas using PCR with the Venor®GeM kit (Minerva Biolabs, Berlin, Germany) and by bovine viral diarrhea virus using immunostaining with an in-house swine anti-BVDV hyperimmune serum kindly provided by the National Reference Center for BVDV (Institute of Veterinary Virology, Vetsuisse Faculty, University of Bern, Switzerland). Cell passages 5 to 8 only were used for the experiments. Bovine cells were routinely seeded 24 h before experiments: 0.8 × 10^6^ cells per 96-well plates (~8.33 × 10^3^ cells/well) (greiner bio-one, Frickenhausen, Germany), 1.5 × 10^6^ cells per 24-well plates (~6.25 × 10^4^ cells/well) (TPP®, Techno Plastic Products AG, Klettgau, Germany) and 1.5 × 10^6^ cells per 6-well plates (~2.5 × 10^5^ cells/well) (TPP®). Twenty-four hours later when the cells were used, there were approximately: 2 × 10^4^ cells/well in 96-well plates (for fluorescence microscopy assays), 7 × 10^4^ cells/well in 24-well plates (for gentamicin and cell entry inhibition assays) and 3 × 10^5^ cells/well in 6-well plates (for TEM assays).

### Turbinate cells infection model and gentamicin protection assay

In order to standardize the in vitro infection model of *M. bovis* using PECT cells, mycoplasma standard curves of concentrations comparing OD_600_ values and 10-fold serial dilutions were performed. Moreover, growth characteristics of *M. bovis* were tested to assess variations among each individual SP4 batch. For all in vitro experiments, mycoplasma cultures were diluted in growth medium to reach the required concentration taking an OD_600_ of 0.1 corresponding to approximately 10^8^ colony-forming units (CFU)/mL. Concentrations were subsequently confirmed by plating 10-fold serial dilutions for CFU enumeration.

*M. bovis* strain JF4278 was selected to study the survival of this bacterium in MEM-Earle medium and in MEM-Earle preincubated with PECT cells either for 24 or 48 h, to confirm the inability of *M. bovis* to grow and survive in MEM-Earle medium and in spent MEM-Earle medium.

Since lack of bacterial cell wall makes mycoplasmas more sensitive to detergents, the efficiency of mechanical lysis of PECT cells was assessed. After growth to confluence, PECT cells were scraped off the growth plates and lysed by pipetting them up and down for a few minutes using tips for 20–200 μL pipettes. Cell viability and morphology were assessed by trypan blue exclusion and by subsequent culture of the lysed PECT cells. Since only 24-well plates were used for mycoplasmas enumeration after cell infection, bovine cells were grown for 24 h in 24-well plates with the same conditions as described above. They were further washed in phosphate-buffered saline (PBS) at pH 7.5 and suspended in 0.5 mL fresh MEM-Earle medium, scraped, lysed and incubated for 10 days at 37 °C with 5% CO_2_.

For the infection assays, SP4 broth cultures of *M. bovis* (Table 1) containing 10^5^ CFU/mL were centrifuged 15 min at 8000 rpm and washed once in PBS at pH 7.5 and mycoplasmas were further suspended in MEM-Earle medium in the same initial volume. PECT cells were infected at a multiplicity of infection (MOI) between 2 and 30. Thereafter, a centrifugation step of 600 × *g* for 5 min was performed. Infected cells were washed at time points 3 and 6 (hours) post infection to carry out the same conditions as for the gentamicin protection assay (see below). After several time points (h): 0, 6, and 54, 10-fold serial dilutions were spotted on SP4 agar plates and incubated. Subsequently, colonies were counted under a stereomicroscope. The assay was performed in triplicates in two independent experiments.

Before starting the gentamicin protection assays, the incubation time with gentamicin necessary to kill all extracellular *M. bovis* at the concentration used for the gentamicin protection assay was evaluated. A concentration of 400 μg/mL gentamicin sulfate (Sigma-Aldrich) was chosen as used in previous studies [17,31]. The bacteria were grown in SP4 broth at the concentration used for infection assays as described above, pelleted by centrifugation, suspended in MEM-Earle medium with addition of a final concentration of 400 μg/mL gentamicin. Ten-fold serial dilutions were spotted on SP4 agar plates at 0, 1 and 3 h to measure the survival time of *M. bovis*. The plates were incubated and CFU/mL were determined.

For the gentamicin protection assay, *M. bovis* strains (Table 1) were grown in SP4 broth until mid-log phase, washed once in PBS and then suspended in MEM-Earle medium to further infect PECT cells in a 24-well plate at an MOI between 2 and 30. Thereafter, the plate was centrifuged 5 min at 600 × *g* and then incubated for 3 h to allow mycoplasmas to infect bovine cells. Cells were washed twice with PBS and suspended in fresh MEM-Earle medium supplemented with 400 μg/mL gentamicin sulfate. The plate was incubated for 3 h (total of 6 h post infection). Thereafter wells were washed three times with PBS, and fresh MEM-Earle medium without gentamicin was added to each well. At different time points (h), 0, 6 and 54 post infection, wells were washed once with PBS and bovine cells were scraped off the growth plates and further mechanically lysed as described above. Samples were taken to measure the quantity of mycoplasmas inside the cells to determine the CFU/well. As controls, assays were performed for all strains without addition of the antibiotic, as well as with PECT cells and mycoplasmas alone. Moreover, to measure the kinetic of the growth of *M. bovis* during the gentamicin protection assay, a growth curve was determined for strain JF4278 as described above for the gentamicin protection assay but including the following time points (h): 0, 3, 6, 30, 54, 78 and 102 post infection. All assays were performed in triplicates in two independent experiments.

During the experiment, cells were visualized by light microscopy. At each time point, cells were fixed with methanol and stained with crystal violet solution (0.75 g crystal violet, 0.25 g NaCl, 1.75 mL formaldehyde in a final volume of 100 mL of 50% ethanol:ddH_2_O).

### Inhibition of endocytic pathways in PECT cells

To investigate the mechanism of entry of *M. bovis* into PECT cells, endocytosis dependent on clathrin-coated vesicles, lipid rafts/caveolin mediated endocytosis and macropinocytosis were individually blocked with several chemical inhibitors (summary in Additional file 1). Formation of clathrin-coated vesicles was inhibited with monodansylcadaverine (MDC) (Sigma-Aldrich), which blocks the assembly of clathrin-coated pits at the plasma membrane, with chlorpromazine (CPZ) (Sigma-Aldrich), a clathrin sequestering agent preventing clathrin recycling in endosomes [37] and with hypertonic sucrose, which removes clathrin lattices from the plasma membrane [38]. Lipid rafts/caveolin based endocytosis occurs in cholesterol rich regions of the cellular plasma membrane [39] and was blocked using methyl-ß-cyclodextrin (MßCD) (Sigma-Aldrich), a substance removing cholesterol from the cell membranes and thereby disturbing the lipid structure [40], nystatin (Sigma-Aldrich), which sequesters cholesterol [37] and simvastatin (Merck Millipore, Darmstadt, Germany), a statin inhibiting the 3-hydroxy-3-methylglutaryl coenzyme A (HMG-CoA) reductase, a rate-limiting enzyme of cholesterol biosynthesis [41]. Macropinocytosis was prevented by treatment with amiloride hydrochloride (Merck Millipore) and 5-(N-Ethyl-N-Isopropyl)-amiloride (EIPA) (Enzo Life Sciences, Lausen, Switzerland), which both block Na^+^/H^+^ exchange and thereby decrease the submembranous intracellular pH. This leads to disturbance of actin remodeling necessary for efficient macropinocytosis [42]. Moreover, cytochalasin D (Sigma-Aldrich), which blocks actin polymerization was tested [43]. Since modulation of the actin cytoskeleton also affects clathrin- (EIPA, cytochalasin D) and caveolae-mediated endocytosis (cytochalasin D) [44], those chemicals are not considered as specific inhibitors of macropinocytosis. For this reason, two additional inhibitors of the phosphoinositide-3 kinase (PI3K), namely wortmannin (Merck Millipore) and LY-294002 (Merck Millipore) were used. These chemicals inhibit macropinocytosis and phagocytosis [45], although it has to be mentioned that they were previously described as possibly interfering with clathrin- and caveolin-mediated endocytosis [46], therefore showing a pleiotropic effect as well.

In a previous study, FACS analysis showed only weak cytotoxicity of inhibitors as 0.2 mM MDC, 25 μM CPZ, 12.5 μg/mL nystatin, 10 mM MßCD and 20 μM EIPA towards PECT cells [47]. To study possible cytotoxic effects of amiloride hydrochloride, simvastatin, sucrose, cytochalasin D, wortmannin and LY-294002 towards PECT cells, chemicals were added to cells at the concentrations defined below, incubated either 3.5 h, or 27 h for simvastatin, at 37 °C supplemented with 5% CO_2_, thereafter stained with crystal violet solution and viewed under the stereomicroscope.

Additionally, the effect of each inhibitor on growth of *M. bovis* strain JF4278 was evaluated. MEM-Earle medium was mixed individually with 25 μM CPZ, 0.2 mM MDC, 10 mM MßCD, 12.5 μg/mL nystatin, 10 μM simvastatin, 5 mM amiloride hydrochloride, 20 μM EIPA, 100 nM wortmannin, 20 μM LY-294002, 0.45 M sucrose, or 5 μg/mL cytochalasin D as previously described [20,37,47-50] and incubated with *M. bovis* strain JF4278 for 3 h at 37 °C supplemented with 5% CO_2_.

**Figure 1:**
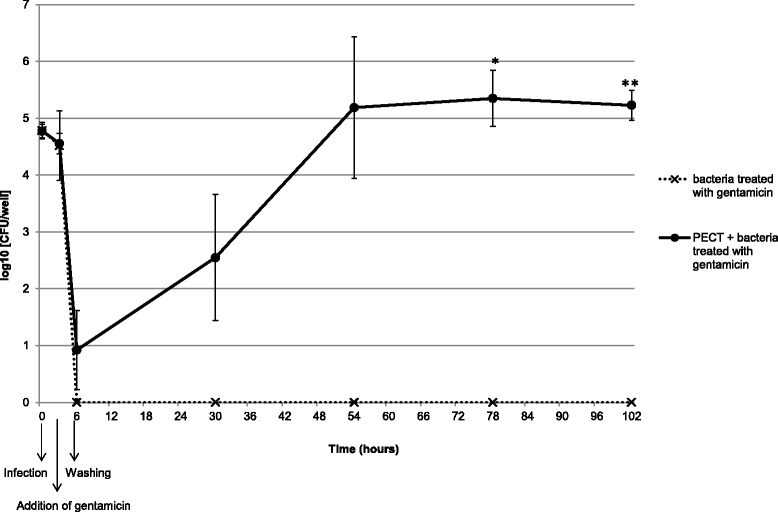
**Intracellular growth curve of** ***M. bovis*** **in PECT cells.** Broken line and crosses: *M. bovis* strain JF4278 with gentamicin. Continuous line and dots: PECT cells and *M. bovis* strain JF4278 with gentamicin. The data shown are the mean values of triplicates of 2 independent experiments and standard deviations of individual measurements are indicated as bars. *P < 0.05, **P < 0.01.

To test the blocking of uptake of *M. bovis*, PECT cells were grown in a 24-well plate to reach confluence and incubated 24 h *prior* to infection with simvastatin or 30 min *prior* to infection for all other inhibitors with the specific concentration described above. *M. bovis* strain JF4278 was grown in SP4 medium and diluted to 10^5^ mycoplasmas per well to infect pretreated PECT cells (no removal of inhibitors) at an MOI between 2 and 30 as used for the gentamicin protection assay. Further steps were performed as described above for the gentamicin protection assay. Cells were mechanically lysed and samples taken at 0 and 10 h after infection. This last time point was chosen to avoid measuring interferences of the inhibitors with intracellular growth as it was previously determined that at this time point after infection, *M. bovis* is at the beginning of the logarithmic growth phase (Figure 1). Ten-fold dilutions were spotted on SP4 agar and *M. bovis* colonies were counted. Negative controls consisted of the infection of untreated eukaryotic cells. Assays were performed in triplicates in two independent experiments.

### Fluorescence microscopy with differential staining

Fluorescence microscopy was performed to distinguish between intra- and extracellular mycoplasmas during cell infection using an antibody independent differential staining [51]. The principle is to label bacteria with 5/6-carboxylfluorescein-succinimidyl ester (NHS-Fluorescein), a molecule derived from fluorescein, which is membrane impermeable and specifically binds to primary amines (lysine side chains) of membrane proteins. Subsequently, bacteria were biotinylated. After infection of eukaryotic cells with labeled and biotinylated bacteria, cells were fixed and extracellular bacteria are stained with streptavidin. This method allows differential staining of intracellular and extracellular bacteria without requiring any antibodies and cell permeabilization.

More specifically, *M. bovis* strain JF4278 was grown until mid-log phase. As negative control and to assess a possible passive internalization of particles into PECT cells, mycoplasma cultures were divided in 2 parts and one part was inactivated by treatment with 3% formaldehyde for 30 min. Mycoplasmal viability was evaluated by culture of 10-fold serial dilutions on SP4 agar plates. *L. monocytogenes* strain JF3263 was used as a positive control for facultative intracellular bacteria and was grown overnight. The next day, several colonies were picked from the plate and transferred into 1 mL MEM-Earle Medium for further processing. Inhibition of *L. monocytogenes* bovine cell invasion was also tested by treatment with 3% formaldehyde for 30 min. Inactivation of *L. monocytogenes* was assessed by plating 10-fold serial dilutions on blood agar plates. Bacterial strains were then washed twice with PBS at pH 7.5 and suspended in 15 μg/mL 5/6-NHS-Fluorescein (Thermo Fisher Scientific) solubilized in PBS. After an incubation period of 15 min at 4 °C on a shaker, succinimidyl-6-(biotinamido) hexanoate (EZ-Link NHS-LC-biotin) (Thermo Fisher Scientific) was added at a final concentration of 0.3 mg/mL in PBS. EZ-link NHS-LC-biotin is membrane permeable and binds to and biotinylates intracellular and membrane primary amines of proteins. The FITC-biotin mixture was then incubated for 30 min at 4 °C, washed three times with PBS and suspended in PBS with 1% albumin.

PECT cells were seeded in 96-well μ-clear plates (greiner bio-one), which minimize background fluorescence, and then infected with the biotinylated and NHS-fluorescein-labeled bacteria at an MOI of 3400 for 6 h for *M. bovis.* This higher MOI was used in order to decrease the time of infection because when mycoplasmas multiply, stains are diluted and fluorescence is no more clearly detectable. In the case of *L. monocytogenes,* an MOI of 55 and infection for 3 h at 37 °C with 5% CO_2_ was performed since at higher MOIs *L. monocytogenes* was cytotoxic to bovine cells. PECT cells alone were used as control. After the incubation period, cells were fixed for 20 min with 4% formaldehyde, washed three times with PBS and blocked for 5 min with PBS complemented with 10% calf serum.

Streptavidin Alexa Fluor® 647 Conjugate (Invitrogen, Life Technologies, Carlsbad, USA) diluted 1:200 in blocking buffer was added and the biotinylated mycoplasmas were successively stained for 45 min at room temperature. Streptavidin is a biotin binding protein, membrane impermeable and therefore only stains extracellular mycoplasmas. Wells were then washed three times with PBS and cell nuclei were stained with a solution of 4′,6-diamidino-2-phenylindole (DAPI dilactate) (Sigma-Aldrich) at a concentration of 1 μg/mL in PBS. Thereafter, cells were stained for 15 min with rhodamine phalloidin (Invitrogen), diluted 1:200 in PBS. Phalloidin selectively binds to F-actin thus visualizing the whole structure of eukaryotic cells. Wells were finally washed three times with PBS and stored at 4 °C in the dark until further processing. To confirm that bacterial staining was correctly performed, *M. bovis* labeled with either NHS-Fluorescein or Streptavidin Alexa 647 alone were seeded in wells without PECT cells, fixed, washed and stored at 4 °C in the dark until analysis.

Cells were visualized using the INCell Analyzer 2000 system (General Electric Healthcare, Glattbrugg, Switzerland). Four to 30 regions per well were selected and images obtained using a wide field epifluorescence microscope with a Nikon objective lens (60x/NA 0.70), at a working distance of 1.8 mm and using the filters for FITC (Excitation filter at 490 nm, Emission filter at 525 nm) for the visualization of the NHS Fluorescein stain, Cy5 (Ex.: 645 nm, Em.: 705 nm) for Streptavidin Alexa 647, DAPI (Ex.: 350 nm Em.: 455 nm) for the DAPI stain and Cy3 (Ex.: 543 nm, Em.: 605 nm) to see the F-actin stained by rhodamine phalloidin. Images were analyzed and merged images were acquired using the INCell Investigator 1.6.2 software (GE Healthcare).

### Fluorescence microscopy using antibodies directed against *M. bovis*

To confirm the strict intracellular multiplication of *M. bovis*, gentamicin treated infected cells (having the same conditions than in the gentamicin protection assay but using 96-well μ-clear plates (greiner bio-one)) as well as untreated controls were fixed with 4% formaldehyde at time point 54 h. Subsequently, two different conditions were used: permeabilized and not permeabilized PECT cells. Briefly, permeabilized cells were treated for 20 min at room temperature with 0.2% triton X-100 in blocking buffer (5% inactivated horse serum in PBS). All cells were further washed three times with PBS. Cells were stained by addition of a mouse monoclonal IgG1 antibody specific to *M. bovis* (Thermo Scientific, Reinach, Switzerland) at a dilution of 1:100 for 90 min at room temperature. Thereafter cells were washed and goat polyclonal anti-mouse secondary IgGs (γ-chain specific) FITC-conjugated (Sigma Aldrich) at a dilution of 1:100 were added for 90 min at room temperature. After washing cells three times with PBS, cell nuclei were stained with DAPI dilactate (Sigma Aldrich) at a dilution of 1:2000 for 15 min followed by staining F-actin using 100 nM rhodamin phalloidin (Lubio Science GmbH, Lucerne, Switzerland) for 30 min at room temperature. Then wells were washed and stored at 4 °C in the dark until analysis with the INCell Analyzer 2000 system (General Electric Healthcare) as described above.

### Transmission electron microscopy

TEM was performed to visualize *M. bovis* during in vitro cell infection. PECT cells were seeded in 6-well plates. Thereafter, cells were infected with strain JF4278 as described above at an MOI of 600 and incubated for 16 h. The negative control consisted of PECT cells alone. After incubation, cells were first washed once with PBS prewarmed to 37 °C and subsequently fixed with 1.5% glutaraldehyde in 0.1 M cacodylate buffer pH 7.4 for 30 min at 37 °C. After three more washes in cacodylate buffer, bacteria were post-fixed with 1% OsO_4_ (Chemie Brunschwig, Basel, Switzerland) in 0.1 M cacodylate buffer for one hour at 4 °C and again washed three times with cacodylate buffer. Thereafter, cells were dehydrated in an ascending ethanol series and embedded in Epon, a mixture of Epoxy embedding resin, dodecenyl succinic anhydride (DDSA) and methyl nadic anhydride (MNA) (Sigma Aldrich). Epon was polymerized for 5 days at 60 °C. Resin blocks were then trimmed and ultrathin sections of 60 nm were obtained with diamond knives (Diatome, Biel, Switzerland) on a Reichert-Jung Ultracut E (Leica, Heerbrugg, Switzerland). Sections were double-stained with 0.5% uranyl acetate for 30 min at 40 °C (Sigma Aldrich, Steinheim, Germany) and 3% lead citrate for 10 min at 20 °C (Laurylab, Saint Fons, France) in an Ultrastain® (Leica, Vienna, Austria) and examined with a Philips CM12 transmission electron microscope (FEI, Eindhoven, The Netherlands) at magnifications ranging from 4400 × to 15 000 ×. Micrographs were captured with a Mega View III camera using the iTEM software (version 5.2; Olympus Soft Imaging Solutions GmbH, Münster, Germany).

### Statistical analysis

*M. bovis* titers (log10 [CFU/well]) measured during infection assays are shown as means ± standard deviations of triplicate values from two independent experiments. For the inhibition of endocytosis assay, all values were normalized to untreated control samples. The significance of differences between individual groups in the assays was calculated with the Student’s *t*-test. Differences among individual strains in the same group were calculated using the Kruskal-Wallis nonparametric ANOVA test and in case of a *p*-value of <0.05, *post hoc* tests were done using Dunn’s multiple comparison test using the software GraphPad Instat™ V2.05 (GraphPad Software Inc., La Jolla, CA, USA).

## Results

### PECT model for *M. bovis* infection

**Figure 2:**
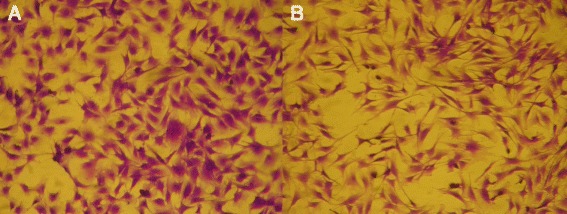
**PECT cells morphology by light microscopy.** Morphological features of PECT cells stained with crystal violet. Panel **A**: PECT cells at passage 8. Panel **B**: PECT cells at passage 10. Magnification 112.5 ×.

Since primary cells were used, we attempted to characterize the type of cells present more in detail. Morphological features of PECT cells were checked by light microscopy and by TEM. Until passage number 8 the majority of the cells were polygonal (epithelial-like), while at passage number 10 most of the cells were fusiform (fibroblast-like) (Figure 2). Moreover in transmission electron micrographs, cells displayed scarce microvilli all along the plasma membrane and didn’t show any sign of polar differentiation. We found no evidence of cilia or of mucous granules. Furthermore, cell population looked morphologically uniform.

**Figure 3:**
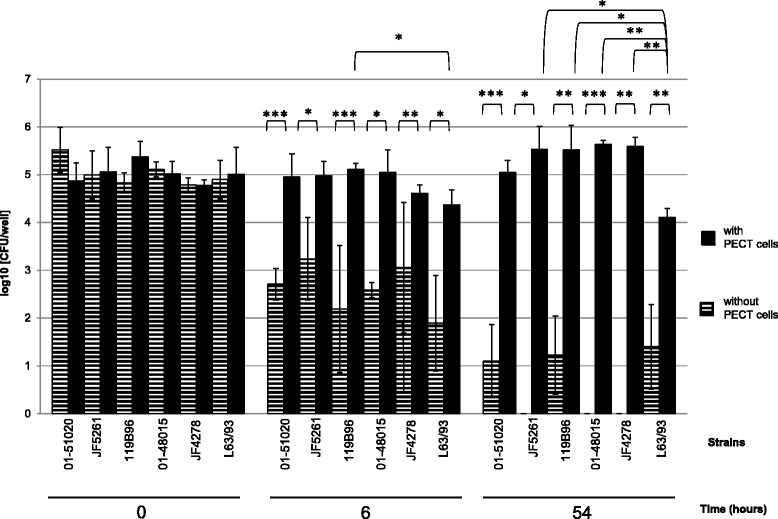
**Infection model of** ***M. bovis*** **with PECT cells.** Time points 0, 6 and 54 h post infection with an MOI between 2 and 30 are shown. Horizontally striped columns correspond to *M. bovis* alone in growth medium, while filled columns correspond to the infection of PECT cells. The data shown are the mean values of triplicates of 2 independent experiments. Standard deviations of individual measurements are indicated as bars. *P < 0.05, **P < 0.01, ***P < 0.001.

None of the *M. bovis* strains used in this study grew in the medium used for eukaryotic cell culture as bacterial concentrations decreased after 6 h incubation in MEM-Earle (Figure 3) and in spent MEM-Earle for strain JF4278 (Additional file 2). Most of the *M. bovis* strains tested lost viability after 54 h incubation in MEM-Earle. Mycoplasma concentrations remained stable in co-culture with PECT cells (MOI between 2 and 30), when cells were not treated with gentamicin, with a slight increase 54 h post infection (Figure 3).

In order to lyse bovine cells without damaging mycoplasmas, mechanical lysis of PECT cells was evaluated. Trypan blue exclusion of lysed PECT cells did not show any viable cells. Subsequent seeding of lysed cells led to less than 10 cell islands per well after 4 days incubation indicating the survival of only very few PECT cells after mechanical lysis and corroborating the effectiveness of this method for further experiments to evaluate the intracellularity of *M. bovis* in eukaryotic cells.

### Invasion and persistence of *M. bovis* in primary bovine epithelial cells

**Figure 4:**
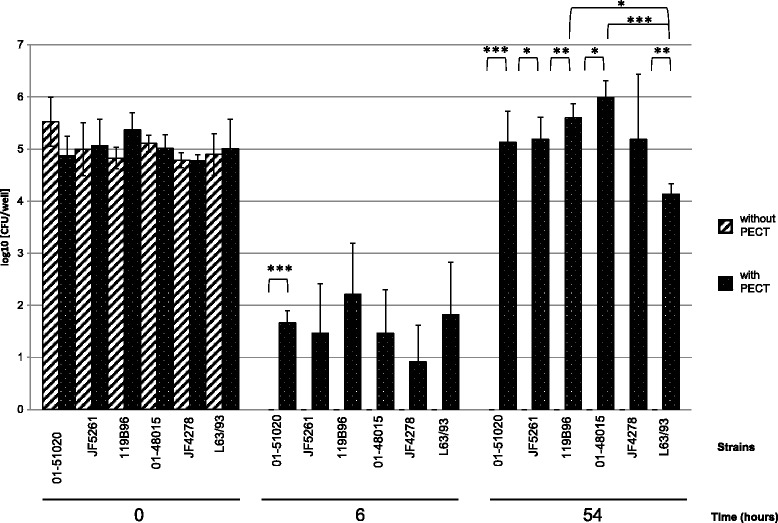
**Gentamicin protection assay.** Time points 0, 6 and 54 h post infection with an MOI between 2 and 30 are shown. Time 6 h post infection corresponds to the end of the gentamicin treatment. Diagonally striped columns correspond to *M. bovis* alone in growth medium supplemented with gentamicin, while dotted columns correspond to the infection of PECT cells with growth medium supplemented with gentamicin. The data shown are the mean values of triplicates of 2 independent experiments. Standard deviations of individual measurements are indicated as bars. *P < 0.05, **P < 0.01, ***P < 0.001.

Infection of PECT cells with *M. bovis* at 10^5^ CFU/0.5 mL, corresponding to an MOI between 2 and 30 and incubated in MEM-Earle medium supplemented with 400 μg/mL gentamicin sulfate, resulted in the death of all *M. bovis* cells after 3 h (Figure 4). After 3 h incubation with gentamicin (6 h post infection), mycoplasma titers in infected PECT cells decreased 10^3^ times (Figure 4). At 48 h post treatment with gentamicin (54 h post infection), values increased to between 10^4^ CFU/well and 10^6^ CFU/well (Figure 4). The intracellular growth curve of strain JF4278 showed that after the logarithmic growth of approximately 10^4^ mycoplasmas, a plateau was reached 78 h post infection (Figure 1). During infection, bovine cells were observed by light microscopy, and were shown to remain attached to the plate. In the controls, mycoplasmas alone without eukaryotic cells immediately died after the gentamicin treatment (Figures 1 and 4).

### *M. bovis* may enter PECT cells *via* a non-classical endocytic pathway

Preliminary tests to evaluate the effect of chemical inhibitors towards growth of *M. bovis* and cytotoxicity towards PECT cells were performed. Growth of mycoplasmas was totally inhibited or decreased by 10 mM MßCD and 5 mM amiloride hydrochloride, but not by 25 μM CPZ, 12.5 μg/mL nystatin, 0.2 mM MDC, 10 μM simvastatin, 20 μM LY-294002, 0.45 M hypertonic sucrose, 5 μg/mL cytochalasin D, 100 nM wortmannin, and 20 μM EIPA (Additional file 1). Consequently MßCD and amiloride hydrochloride were excluded from further experiments. Moreover, incubation of PECT cells with 10 μM simvastatin and 20 μM LY-294002 for 3 h showed no cytotoxic activity, whereas, incubation with 0.45 M sucrose, 5 μg/mL cytochalasin D, 5 mM amiloride hydrochloride and 100 nM wortmannin were associated with PECT cell death after 3 h incubation (Additional file 1). Therefore these four chemicals were excluded from the experiment.

**Figure 5:**
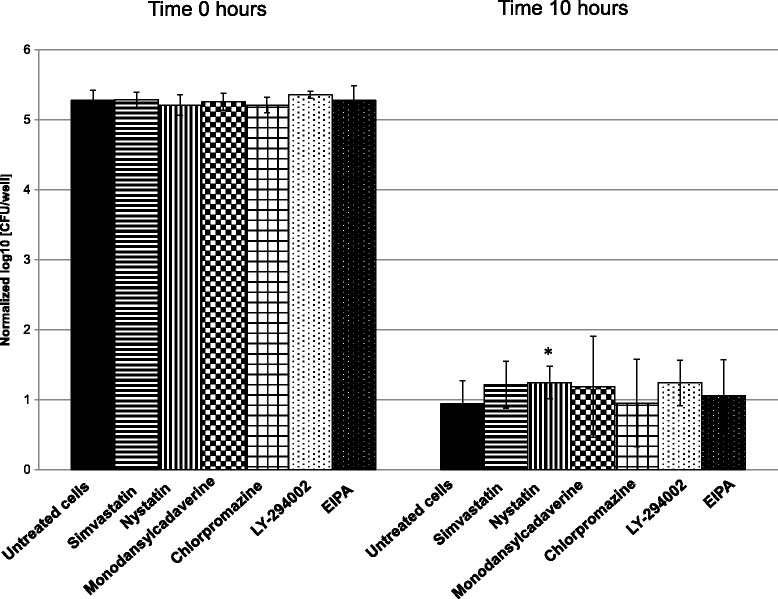
**Inhibition of** ***M. bovis*** **entry in PECT cells by chemical treatment.** The data shown are the mean values of the triplicates of 2 independent experiments at time 0 and 10 h using an MOI between 2 and 30, the latter 4 h after the gentamicin incubation time. Standard deviations of the individual measurements are indicated as bars. *P < 0.05.

PECT cells pretreated with drugs to block bacterial cell entry in eukaryotic cells showed no clear inhibition of *M. bovis* invasion 10 h post infection (Figure 5). However, it has to be noted that results obtained after cell treatment with MDC and CPZ, both inhibitors of clathrin-mediated endocytosis revealed inhibition in certain assays, but showed variability between individual assays (Figure 5).

### Extra- and intracellular *M. bovis* visualization by fluorescence microscopy

**Figure 6:**
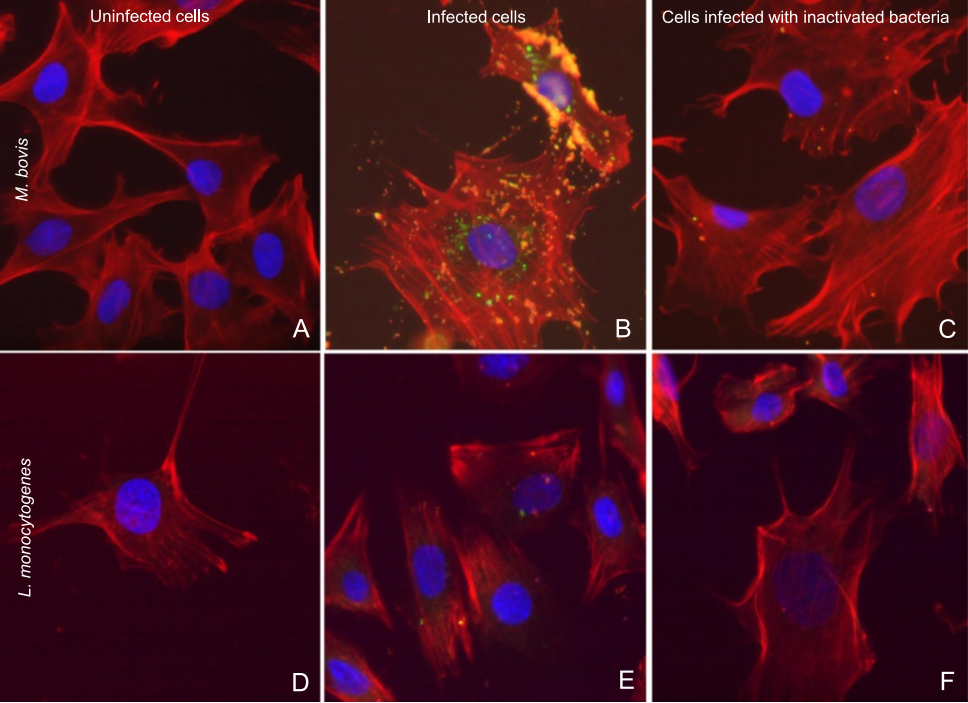
**Differential fluorescence microscopy of PECT cells infection with** ***M. bovis*** **and** ***L. monocytogenes.*** Panels **A**, **B** and **C**: infection with *M. bovis* at an MOI of 3400, 6 h post infection. Panels **D**, **E** and **F**: infection with *L. monocytogenes* at an MOI of 55, 3 h post infection. Panels **A** and **D**: negative controls. Panels **B** and **E**: infected cells. Panels **C** and **F**: cells infected with formaldehyde-inactivated bacteria. Nuclei are stained in blue, F-actin is stained in red, intracellular bacteria are stained in green and extracellular bacteria are stained in yellow-orange. Merged images. Magnification 600 ×.

Differential staining was performed to distinguish between intracellular and extracellular *M. bovis* by fluorescence microscopy. Intracellular *M. bovis* were only stained by NHS-Fluorescein and visualized in green, while extracellular mycoplasmas were stained with NHS-Fluorescein and Streptavidin-Alexa 647 and appeared in yellow-orange (Figure 6; panel B). Cell nuclei were stained with DAPI (blue) and F-Actin was visualized with rhodamine phalloidin (red) (Figure 6, panels A, B and C). Many extracellular *M. bovis* clusters were visible surrounding or attaching to the PECT cells since no gentamicin was added in this experiment (Figure 6, panel B). Intracellular mycoplasmas were visible as clusters within PECT cells but did not show any clear localization in a specific cell compartment (Figure 6, panel B). In the control, using formaldehyde-inactivated mycoplasmas, adhesion and invasion were almost totally inhibited (Figure 6, panel C).

*L. monocytogenes* used as positive control confirmed the efficacy of this method to distinguish between intracellular and extracellular bacteria (Figure 6, panel E). In this case only one *L. monocytogenes* per eukaryotic cell was observed (Figure 6, panel E) but using a low MOI because of the cytotoxicity observed towards PECT cells. Moreover, using formaldehyde-inactivated listerias, adhesion and invasion were inhibited (Figure 6, panel F).

**Figure 7:**
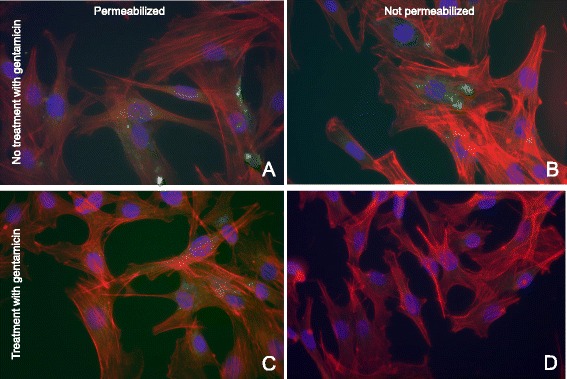
**Fluorescence microscopy of the gentamicin protection assay with permeabilized and not permeabilized cells using antibodies directed against** ***M. bovis*** **.** MOI of 10; 54 hours post infection. Panel **A**: Permeabilized PECT cells without gentamicin treatment infected with *M. bovis* JF4278. Mycoplasmas are visible intra- and extracellularly. Panel **B**: Non permeabilized PECT cells without gentamicin treatment infected with *M. bovis* JF4278. Only extracellular mycoplasmas are visible. Panel **C**: Permeabilized PECT cells with gentamicin treatment infected with *M. bovis* JF4278. Only intracellular mycoplasmas are visible. Panel **D**: Not permeabilized PECT cells with gentamicin treatment infected with *M. bovis* JF4278. No mycoplasmas are visible. Nuclei are stained in blue, F-actin is stained in red, mycoplasmas are stained in green. Merged images. Magnification 600 ×.

Moreover, fluorescence microscopy using mouse monoclonal antibodies directed against *M. bovis* was used to assess intracellular replication. When infection was performed without treatment with gentamicin and cells were stained after cell permeabilization, intracellular and extracellular *M. bovis* were visible (Figure 7, panel A), while when cells were not permeabilized only extracellular mycoplasmas were detected (Figure 7, panel B). Moreover, when infection was followed by treatment with gentamicin, *M. bovis* could be observed intracellularly when cells were permeabilized (Figure 7, panel C) but not if cells were stained without a previous step of permeabilization (Figure 7, panel D).

### Ultrastructural characterization of internalization of *M. bovis*

In the micrographs, PECT cells displayed scarce microvilli all along the plasma membrane and didn’t show any sign of polar differentiation. We found no evidence of cilia or of mucous granules. Thus, as the cells were lacking such specific features, morphology was closest to basal or principal cells. Moreover, cell population looked uniform.

**Figure 8:**
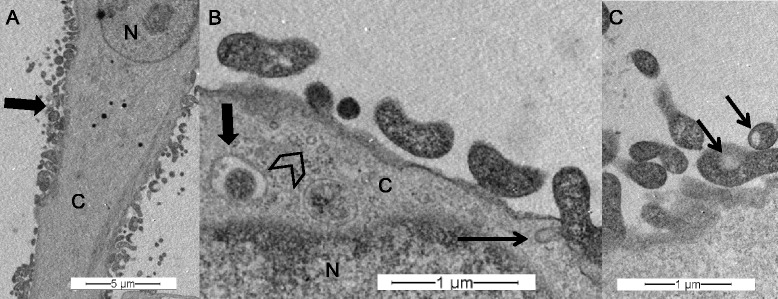
**Transmission electron microscopy of PECT cells infected with** ***M. bovis*** **.** MOI of 600, 16 h post infection. Panel **A**: *M. bovis* infected PECT cell, *M. bovis* clusters (bold arrow). Panel **B**: PECT cell invagination (thin arrow), intracellular vesicles (arrowhead), intracellular, membrane-bound *M. bovis* (bold arrow). Panel **C**: electron-lucent space in mycoplasmal cytoplasm (thin arrow). N: PECT cell nucleus; C: PECT cell cytoplasm.

In TEM experiments, *M. bovis* cells appeared as pleomorphic but mostly oval to elongated structures of 0.8 μm to 2 μm length and 0.1 μm to 0.3 μm in width, showing varying electron densities (Figure 8, panels A and B). At 16 h post infection, bacteria were mostly found in close proximity or adhering to the cells. Intracellular *M. bovis* were observed in vesicle-like structures having a second membrane layer, the plasmalemma of the mycoplasmas being occasionally separated from the vesicle’s membrane by an electron-lucent space (Figure 8, panel B). Moreover, several mycoplasmas were seen with electron-lucent spaces in their cytoplasm (Figure 8, panel C). Multiple invaginations at the cell surface, 50 nm-100 nm in diameter with openings of approximately 50 nm and various submembranous vesicles of 50–100 nm in diameter were seen in PECT cells where mycoplasmas adhered to bovine cells (Figure 8, panel B).

## Discussion

The present study demonstrates the ability of *M. bovis* to invade, persist and multiply in PECT cells. These cells were chosen instead of characterized cell lines in order to observe mechanisms that are the closest to the in vivo situation. Possible cell types from the respiratory epithelium include basal cells, principal cells, ciliated cells and goblet cells. Moreover, a minimal contamination with fibroblasts from the lamina propria cannot be totally excluded. As primary cells contain mixed cell types, cells were checked for morphological features by light microscopy and by TEM. Until passage number 8, the majority of the cells were polygonal (epithelial-like), while at passage number 10 most of the cells were fusiform (fibroblast-like) (Figure 2). For this reason, only cells at passage numbers between 5 and 8 were used for all the experiments. Moreover as shown by TEM, the cell morphology was closest to basal or principal cells and was fairly uniform. At low MOI, between 2 and 30, all *M. bovis* strains survived within PECT cells and were able to multiply, reaching 10^3^ until 10^4^ times the initial CFU values 48 h after removal of gentamicin. This corresponds to an average generation time of at least 5.7 h, which is about twice the generation time under axenic growth conditions. These results suggest that *M. bovis* multiplies inside PECT cells since *M. bovis* is not able to survive or multiply in MEM-Earle or in spent MEM-Earle media. Gentamicin was previously shown to have a limited penetration through membranes into cells [52] but some leakage of this antimicrobial substance inside PECT cells cannot be excluded especially at the high concentration of 400 μg/mL used for the gentamicin protection assay. For this reason, treatment was limited to 3 h and after killing of all extracellular mycoplasmas, gentamicin was not added anymore. Interestingly, *M. bovis* showed no apparent cytotoxicity towards PECT cells and the latter even continued to multiply during long infection periods of 78 h.

The ability of *M. bovis* to invade and persist in PECT cells raised the question of the mechanism used to enter epithelial cells. The approach chosen was to block different mechanisms of bacterial cell entry by pretreatment with specific inhibiting drugs. None of the chemicals tested under the conditions used in this infection model showed a clear inhibition of *M. bovis* entry in PECT cells. However, MDC and CPZ demonstrated a high variability of inhibition of entry of *M. bovis* in PECT cells, although with variable effects in between experiments, blocking almost 80% of mycoplasmal cell entry in an individual experiment. Endocytosis comprises complex mechanisms leading to membranes modifications and cytoskeleton rearrangements [53] but our findings suggest that *M. bovis* may invade PECT cells via another endocytic pathway than those tested. However, we cannot exclude that clathrin-mediated endocytosis is not also involved. This hypothesis could explain the inconsistent blocking of entry with MDC and CPZ through the switching to another mechanism of *M. bovis* entry in PECT cells. MDC and CPZ are known to indirectly impact actin dynamics; MDC via inhibition of transglutaminases and CPZ through inhibition of phospholipase C [54]. These chemicals may also potentially interfere to a various extent with an alternative mechanism of cell entry requiring actin assembly and dynamics. Another explanation is that this cell model is based on primary cells. It cannot be excluded that a variation in the proportion of the type of cells present between the two experiments influenced the results. Previous studies in other *Mycoplasma* sp. suggested a role of cytoskeleton rearrangements [20,55,56] and recently, Fürnkranz et al. observed a slight decrease in cell invasion after cholesterol depletion of HeLa cell membranes [54]. In contrast, we didn’t observe any inhibition of cell entry by blocking lipid rafts/caveolin based endocytosis, and results were consistent concerning nystatin.

The differential staining method used for fluorescence microscopy [51] was advantageous since no specific antibodies were required to visualize extra- and intracellularly located *M. bovis*. However, high MOIs should be used for this experiment because bacterial replication dilutes the dyes resulting in less detectable fluorescence. This method was previously shown to not interfere with invasiveness of *Neisseria gonorrhoeae* and *Staphylococcus aureus* [51]. It has to be mentioned that even at high MOIs, *M. bovis* was not detected inside each individual bovine turbinate cell. However numerous clusters of mycoplasmas adhering to the plasma membrane of PECT cells were seen. Intracellularly, *M. bovis* appeared in clusters with no apparent localization in specific cellular compartments as it was observed for *M. gallisepticum* in HeLa-229 cells and chicken embryo fibroblasts (CEF) by double immunofluorescence microscopy [20] or *M. pneumoniae* in normal human bronchial epithelial cells by immunofluorescence microscopy [57]. *M. synoviae* is spread throughout the cytoplasm but is also observed in clusters around the nuclei in the human epithelial cell line HEp-2 as investigated by confocal laser scanning microscopy [58] and *M. genitalium* strains G37 and 1019 V are found peri-, and intranuclearly in HeLa and EM42 cells using immunofluorescence and confocal microscopy [59]. *M. bovis* strain Mb1, invading different populations of PBMCs, displayed different intracellular patterns depending on the individual cell population or the time of infection [31]. Moreover, fluorescent staining of *M. bovis* without prior permeabilization of turbinate cells confirmed the intracellular replication of mycoplasmas. Interestingly, formaldehyde-inactivated *M. bovis* did not invade PECT cells, and no adherence to bovine cells was observed but for residual spots probably due to the high concentration of *M. bovis* used in this experiment. The control using formaldehyde-inactivated *L. monocytogenes* did not show specific spots. However, this variation can be due to the very low MOI used for *L. monocytogenes* because of its high cytotoxicity toward PECT cells. Adhesion is necessary for further cell invasion but the fact that inactivated *M. bovis* are not able to invade PECT cells would suggest the involvement of an active mechanism.

Morphologically, TEM experiments revealed *M. bovis* as oval to elongated electron dense structures of 0.8 μm to 2 μm length and 0.2 μm to 0.3 μm width in agreement with previous descriptions from Maeda et al. [32]. Kleinschmidt et al. [22] reported *M. bovis* to be round to oval or pleomorphic with a diameter of 0.45-0.80 μm and sometimes found the microorganisms within the cytoplasm of phagocytes, in phagosomes and/or phagolysosomes but not in bronchial epithelial cells by TEM of lung tissue sections. This raises the question of different *M. bovis*-cell interactions dependent on host cells. In the present study, multiple mycoplasmas were seen with electron lucent spaces in their cytoplasm, varying in size, as already described by Hirth et al. [60]. However, the lucent spaces were rather small, approximately 150 nm in diameter and poorly defined. Sub-membrane vesicles of 50–100 nm in diameter were observed in PECT cells. The intracellular behavior of some other *Mycoplasma* species was already studied more intensively by TEM and some comparisons among differences or similarities among *Mycoplasma* species can be highlighted. *M. suis* strain 08/07 has been observed inside porcine RBCs in intraerythrocytic vacuoles or free in the cytoplasm [16]. These features were also observed by Vancini and Benchimol, who investigated the ultrastructural characteristics of *M. hominis* strain ATCC 23114 in *Trichomonas vaginalis* and found them inside vacuoles, in close proximity to, or even fused with vacuolar membranes, or free inside the cytosol [18]. In contrast, intracellular *M. genitalium* strains G37 and M2300 were seen in vacuoles throughout the cytosol but mainly in a perinuclear location [61]. Extracellular *M. genitalium* was also observed attached to the plasma membrane of vaginal epithelial cells. In those attachment regions, the mycoplasmas develop polarized electron-dense core structures similar to the electron-dense core seen in terminal tip structures of *M. genitalium* [61].

In summary, *M. bovis* is able to invade, persist and multiply in PECT cells. The intracellular survival of *M. bovis* could represent a protective niche for this bacterium to escape both the host’s immune defense as well as therapy of affected animals with antibiotics. Moreover, the intracellular survival of *M. bovis* can lead to healthy carrier animals and thus to a more widespread transmission of the pathogen within cattle herds.

## Additional files

Additional file 1:
**Inhibitors used to block the different endocytosis pathways.** Table summarizing the inhibitor drugs used to block *M. bovis* entry in PECT cells. Additional file 2:
**Growth curve of strain JF4278 in fresh and spent MEM-Earle medium.** Growth curve showing the survival of *M. bovis* in fresh and spent medium used for eukaryotic cell culture.

## References

[1] Stipkovits L, Rosengarten R, Frey J (1999). Mycoplasmas of ruminants: pathogenicity, diagnostics, epidemiology and molecular genetics.

[2] Caswell JL, Archambault M (2007). *Mycoplasma bovis* pneumonia in cattle. Anim Health Res Rev.

[3] Gonzalez RN, Wilson DJ (2003). Mycoplasmal mastitis in dairy herds. Vet Clin North Am Food Anim Pract.

[4] Pfützner H, Sachse K (1996). *Mycoplasma bovis* as an agent of mastitis, pneumonia, arthritis and genital disorders in cattle. Rev Sci Tech.

[5] Walz PH, Mullaney TP, Render JA, Walker RD, Mosser T, Baker JC (1997). Otitis media in preweaned Holstein dairy calves in Michigan due to *Mycoplasma bovis*. J Vet Diagn Invest.

[6] Vanden Bush TJ, Rosenbusch RF (2003). Characterization of the immune response to *Mycoplasma bovis* lung infection. Vet Immunol Immunopathol.

[7] Ayling RD, Baker SE, Peek ML, Simon AJ, Nicholas RA (2000). Comparison of in vitro activity of danofloxacin, florfenicol, oxytetracycline, spectinomycin and tilmicosin against recent field isolates of *Mycoplasma bovis*. Vet Rec.

[8] Nicholas RAJ, Ayling RD (2003). *Mycoplasma bovis*: disease, diagnosis, and control. Res Vet Sci.

[9] Lysnyansky I, Sachse K, Rosenbusch R, Levisohn S, Yogev D (1999). The *vsp* locus of *Mycoplasma bovis*: gene organization and structural features. J Bacteriol.

[10] McAuliffe L, Ellis RJ, Miles K, Ayling RD, Nicholas RA (2006). Biofilm formation by mycoplasma species and its role in environmental persistence and survival. Microbiology.

[11] Razin S, Jacobs E (1992). Mycoplasma adhesion. J Gen Microbiol.

[12] Rottem S (2003). Interaction of mycoplasmas with host cells. Physiol Rev.

[13] Lo SC, Hayes MM, Kotani H, Pierce PF, Wear DJ, Newton PB, Tully JG, Shih JW (1993). Adhesion onto and invasion into mammalian cells by *Mycoplasma penetrans*: a newly isolated mycoplasma from patients with AIDS. Mod Pathol.

[14] Díaz-García FJ, Herrera-Mendoza AP, Giono-Cerezo S, Guerra-Infante FM (2006). *Mycoplasma hominis* attaches to and locates intracellularly in human spermatozoa. Hum Reprod.

[15] Dusanic D, Bercic RL, Cizelj I, Salmic S, Narat M, Bencina D (2009). *Mycoplasma synoviae* invades non-phagocytic chicken cells in vitro. Vet Microbiol.

[16] Groebel K, Hoelzle K, Wittenbrink MM, Ziegler U, Hoelzle LE (2009). *Mycoplasma suis* invades porcine erythrocytes. Infect Immun.

[17] Hegde S, Hegde S, Spergser J, Brunthaler R, Rosengarten R, Chopra-Dewasthaly R (2014). In vitro and in vivo cell invasion and systemic spreading of *Mycoplasma agalactiae* in the sheep infection model. Int J Med Microbiol.

[18] Vancini RG, Benchimol M (2008). Entry and intracellular location of *Mycoplasma hominis* in *Trichomonas vaginalis*. Arch Microbiol.

[19] Vogl G, Plaickner A, Szathmary S, Stipkovits L, Rosengarten R, Szostak MP (2008). *Mycoplasma gallisepticum* invades chicken erythrocytes during infection. Infect Immun.

[20] Winner F, Rosengarten R, Citti C (2000). In vitro cell invasion of *Mycoplasma gallisepticum*. Infect Immun.

[21] Jacobsen B, Hermeyer K, Jechlinger W, Zimmermann M, Spergser J, Rosengarten R, Hewicker-Trautwein M (2010). In situ hybridization for the detection of *Mycoplasma bovis* in paraffin-embedded lung tissue from experimentally infected calves. J Vet Diagn Invest.

[22] Kleinschmidt S, Spergser J, Rosengarten R, Hewicker-Trautwein M (2013). Long-term survival of *Mycoplasma bovis* in necrotic lesions and in phagocytic cells as demonstrated by transmission and immunogold electron microscopy in lung tissue from experimentally infected calves. Vet Microbiol.

[23] Sachse K, Grajetzki C, Rosengarten R, Hanel I, Heller M, Pfutzner H (1996). Mechanisms and factors involved in *Mycoplasma bovis* adhesion to host cells. Zentralbl Bakteriol.

[24] Sachse K, Helbig JH, Lysnyansky I, Grajetzki C, Muller W, Jacobs E, Yogev D (2000). Epitope mapping of immunogenic and adhesive structures in repetitive domains of *Mycoplasma bovis* variable surface lipoproteins. Infect Immun.

[25] Sachse K, Pfutzner H, Heller M, Hanel I (1993). Inhibition of *Mycoplasma bovis* cytadherence by a monoclonal antibody and various carbohydrate substances. Vet Microbiol.

[26] Song Z, Li Y, Liu Y, Xin J, Zou X, Sun W (2012). a-Enolase, an adhesion-related factor of *Mycoplasma bovis*. PLoS One.

[27] Thomas A, Leprince P, Dizier I, Ball H, Gevaert K, Van Damme J, Mainil J, Linden A (2005). Identification by two-dimensional electrophoresis of a new adhesin expressed by a low-passaged strain of *Mycoplasma bovis*. Res Microbiol.

[28] Thomas A, Sachse K, Dizier I, Grajetzki C, Farnir F, Mainil JG, Linden A (2003). Adherence to various host cell lines of *Mycoplasma bovis* strains differing in pathogenic and cultural features. Vet Microbiol.

[29] Thomas A, Sachse K, Farnir F, Dizier I, Mainil J, Linden A (2003). Adherence of *Mycoplasma bovis* to bovine bronchial epithelial cells. Microb Pathog.

[30] Thomas LH, Howard CJ, Parsons KR, Anger HS (1987). Growth of *Mycoplasma bovis* in organ cultures of bovine foetal trachea and comparison with *Mycoplasma dispar*. Vet Microbiol.

[31] van der Merwe J, Prysliak T, Perez-Casal J (2010). Invasion of bovine peripheral blood mononuclear cells and erythrocytes by *Mycoplasma bovis*. Infect Immun.

[32] Maeda T, Shibahara T, Kimura K, Wada Y, Sato K, Imada Y, Ishikawa Y, Kadota K (2003). *Mycoplasma bovis*-associated suppurative otitis media and pneumonia in bull calves. J Comp Pathol.

[33] Freundt EA, Razin S, Tully JG (1983). Culture media for classic mycoplasmas. Methods in mycoplasmology, Volume 1.

[34] Charpentier E, Courvalin P (1997). Emergence of the trimethoprim resistance gene *dfrD* in *Listeria monocytogenes* BM4293. Antimicrob Agents Chemother.

[35] Pilo P, Vilei EM, Peterhans E, Bonvin-Klotz L, Stoffel MH, Dobbelaere D, Frey J (2005). A metabolic enzyme as a primary virulence factor of *Mycoplasma mycoides* subsp. *mycoides* Small Colony. J Bacteriol.

[36] Schweizer M, Peterhans E (1999). Oxidative stress in cells infected with bovine viral diarrhoea virus: a crucial step in the induction of apoptosis. J Gen Virol.

[37] Law HT, Lin AE, Kim Y, Quach B, Nano FE, Guttman JA (2011). *Francisella tularensis* uses cholesterol and clathrin-based endocytic mechanisms to invade hepatocytes. Sci Rep.

[38] Hansen SH, Sandvig K, van Deurs B (1993). Clathrin and HA2 adaptors: effects of potassium depletion, hypertonic medium, and cytosol acidification. J Cell Biol.

[39] Fielding CJ, Fielding PE (2003). Relationship between cholesterol trafficking and signaling in rafts and caveolae. Biochim Biophys Acta.

[40] Kilsdonk EP, Yancey PG, Stoudt GW, Bangerter FW, Johnson WJ, Phillips MC, Rothblat GH (1995). Cellular cholesterol efflux mediated by cyclodextrins. J Biol Chem.

[41] Liao JK, Laufs U (2005). Pleiotropic effects of statins. Annu Rev Pharmacol Toxicol.

[42] Koivusalo M, Welch C, Hayashi H, Scott CC, Kim M, Alexander T, Touret N, Hahn KM, Grinstein S (2010). Amiloride inhibits macropinocytosis by lowering submembranous pH and preventing Rac1 and Cdc42 signaling. J Cell Biol.

[43] Peterson JR, Mitchison TJ (2002). Small molecules, big impact: a history of chemical inhibitors and the cytoskeleton. Chem Biol.

[44] Ivanov AI (2008). Pharmacological inhibition of endocytic pathways: is it specific enough to be useful?. Methods Mol Biol.

[45] Araki N, Johnson MT, Swanson JA (1996). A role for phosphoinositide 3-kinase in the completion of macropinocytosis and phagocytosis by macrophages. J Cell Biol.

[46] Jess TJ, Belham CM, Thomson FJ, Scott PH, Plevin RJ, Gould GW (1996). Phosphatidylinositol 3′-kinase, but not p70 ribosomal S6 kinase, is involved in membrane protein recycling: wortmannin inhibits glucose transport and downregulates cell-surface transferrin receptor numbers independently of any effect on fluid-phase endocytosis in fibroblasts. Cell Signal.

[47] Zürcher C, Sauter KS, Mathys V, Wyss F, Schweizer M (2014). Prolonged activity of the pestiviral RNase Erns as an interferon antagonist after uptake by clathrin-mediated endocytosis. J Virol.

[48] Amyere M, Payrastre B, Krause U, Van Der Smissen P, Veithen A, Courtoy PJ (2000). Constitutive macropinocytosis in oncogene-transformed fibroblasts depends on sequential permanent activation of phosphoinositide 3-kinase and phospholipase C. Mol Biol Cell.

[49] Qaddoumi MG, Gukasyan HJ, Davda J, Labhasetwar V, Kim KJ, Lee VH (2003). Clathrin and caveolin-1 expression in primary pigmented rabbit conjunctival epithelial cells: role in PLGA nanoparticle endocytosis. Mol Vis.

[50] Slevogt H, Seybold J, Tiwari KN, Hocke AC, Jonatat C, Dietel S, Hippenstiel S, Singer BB, Bachmann S, Suttorp N, Opitz B (2007). *Moraxella catarrhalis* is internalized in respiratory epithelial cells by a trigger-like mechanism and initiates a TLR2- and partly NOD1-dependent inflammatory immune response. Cell Microbiol.

[51] Agerer F, Waeckerle S, Hauck CR (2004). Microscopic quantification of bacterial invasion by a novel antibody-independent staining method. J Microbiol Methods.

[52] Elsinghorst EA (1994). Measurement of invasion by gentamicin resistance. Methods Enzymol.

[53] Lin AE, Guttman JA (2010). Hijacking the endocytic machinery by microbial pathogens. Protoplasma.

[54] Fürnkranz U, Siebert-Gulle K, Rosengarten R, Szostak MP (2013). Factors influencing the cell adhesion and invasion capacity of *Mycoplasma gallisepticum*. Acta Vet Scand.

[55] Andreev J, Borovsky Z, Rosenshine I, Rottem S (1995). Invasion of HeLa cells by *Mycoplasma penetrans* and the induction of tyrosine phosphorylation of a 145-kDa host cell protein. FEMS Microbiol Lett.

[56] Borovsky Z, Tarshis M, Zhang P, Rottem S (1998). Protein kinase C activation and vacuolation in HeLa cells invaded by *Mycoplasma penetrans*. J Med Microbiol.

[57] Prince OA, Krunkosky TM, Krause DC (2014). In vitro spatial and temporal analysis of *Mycoplasma pneumoniae* colonization of human airway epithelium. Infect Immun.

[58] Buim MR, Buzinhani M, Yamaguti M, Oliveira RC, Mettifogo E, Ueno PM, Timenetsky J, Santelli GM, Ferreira AJ (2011). *Mycoplasma synoviae* cell invasion: elucidation of the *Mycoplasma* pathogenesis in chicken. Comp Immunol Microbiol Infect Dis.

[59] Ueno PM, Timenetsky J, Centonze VE, Wewer JJ, Cagle M, Stein MA, Krishnan M, Baseman JB (2008). Interaction of *Mycoplasma genitalium* with host cells: evidence for nuclear localization. Microbiology.

[60] Hirth RS, Tourtellotte ME, Nielsen SW (1970). Cytopathic effects and ultrastructure of *Mycoplasma agalactiae* var. *bovis* (Donetta strain). Infect Immun.

[61] McGowin CL, Popov VL, Pyles RB (2009). Intracellular *Mycoplasma genitalium* infection of human vaginal and cervical epithelial cells elicits distinct patterns of inflammatory cytokine secretion and provides a possible survival niche against macrophage-mediated killing. BMC Microbiol.

[62] Khan LA, Miles RJ, Nicholas RA (2005). Hydrogen peroxide production by *Mycoplasma bovis* and *Mycoplasma agalactiae* and effect of *in vitro* passage on a *Mycoplasma bovis* strain producing high levels of H_2_O_2_. Vet Res Commun.

[63] Aebi M, Bodmer M, Frey J, Pilo P (2012). Herd-specific strains of *Mycoplasma bovis* in outbreaks of mycoplasmal mastitis and pneumonia. Vet Microbiol.

[64] Gagea MI, Bateman KG, Shanahan RA, van Dreumel T, McEwen BJ, Carman S, Archambault M, Caswell JL (2006). Naturally occurring *Mycoplasma bovis*-associated pneumonia and polyarthritis in feedlot beef calves. J Vet Diagn Invest.

