# Supplementary material for: Invasion and persistence of Mycoplasma bovis in embryonic calf turbinate cells
Source: Vet Res. 2015 May 15;46(1):53. doi: 10.1186/s13567-015-0194-z (PMC4432498; doi:10.1186/s13567-015-0194-z)
Supplement: Additional file 1: — Inhibitors used to block the different endocytosis pathways. Table summarizing the inhibitor drugs used to block M. bovis entry in PECT cells. [file 13567_2015_194_MOESM1_ESM.docx]

**Additional File 1 Inhibitors used to block the different endocytosis pathways.**

| Inhibitor (concentration) | Endocytosis pathway | Mechanism | Effect on PECT cells | Effect on *M. bovis* |
| --- | --- | --- | --- | --- |
| Monodansylcadaverine (MDC, 0.2 mM) | Clathrin-mediated endocytosis | blocks the assembly of clathrin-coated pits at the plasma membrane | no | no |
| Chlorpromazine (CPZ, 25 µM) | Clathrin-mediated endocytosis | clathrin sequestering agent preventing clathrin recycling in endosomes | no | no |
| Hypertonic sucrose (0.45 M) | Clathrin-mediated endocytosis | removes clathrin lattices from the plasma membrane | yes | no |
| Methyl-ß-cyclodextrin (MßCD, 10 mM) | Lipid rafts / caveolin-mediated endocytosis | removes cholesterol from the cell membranes and thereby disturbs the lipid structure | no | yes |
| Nystatin (12.5 µg/mL) | Lipid rafts / caveolin-mediated endocytosis | sequesters cholesterol | no | no |
| Simvastatin (10 µM) | Lipid rafts / caveolin-mediated endocytosis | inhibits the 3-hydroxy-3-methylglutaryl coenzyme A (HMG-CoA) reductase, a rate-limiting enzyme in cholesterol biosynthesis | no | no |
| Amiloride hydrochloride (5 mM) | Macropinocytosis | blocks Na^+^/H^+^ exchange and thereby decrease the submembranous intercellular pH, leading to disturbance of actin remodeling necessary for efficient macropinocytosis | yes | yes |
| 5-(N-Ethyl-N-Isopropyl)-amiloride (EIPA, 20 µM) | Macropinocytosis | blocks Na^+^/H^+^ exchange and thereby decrease the submembranous intercellular pH, leading to disturbance of actin remodeling necessary for efficient macropinocytosis | no | no |
| Cytochalasin D (5 µg/mL) | Macropinocytosis | blocks actin polymerization | yes | no |
| Wortmannin (100 nM) | Macropinocytosis and phagocytosis | inhibitor of phosphoinositide-3 kinase (PI3K) | yes | no |
| LY-294002 (20 µM) | Macropinocytosis and phagocytosis | inhibitor of phosphoinositide-3 kinase (PI3K) | no | no |
