# Supplementary figures and images for: Invasion and persistence of Mycoplasma bovis in embryonic calf turbinate cells
Source: Vet Res. 2015 May 15;46(1):53. doi: 10.1186/s13567-015-0194-z (PMC4432498; doi:10.1186/s13567-015-0194-z)

## Slide 1
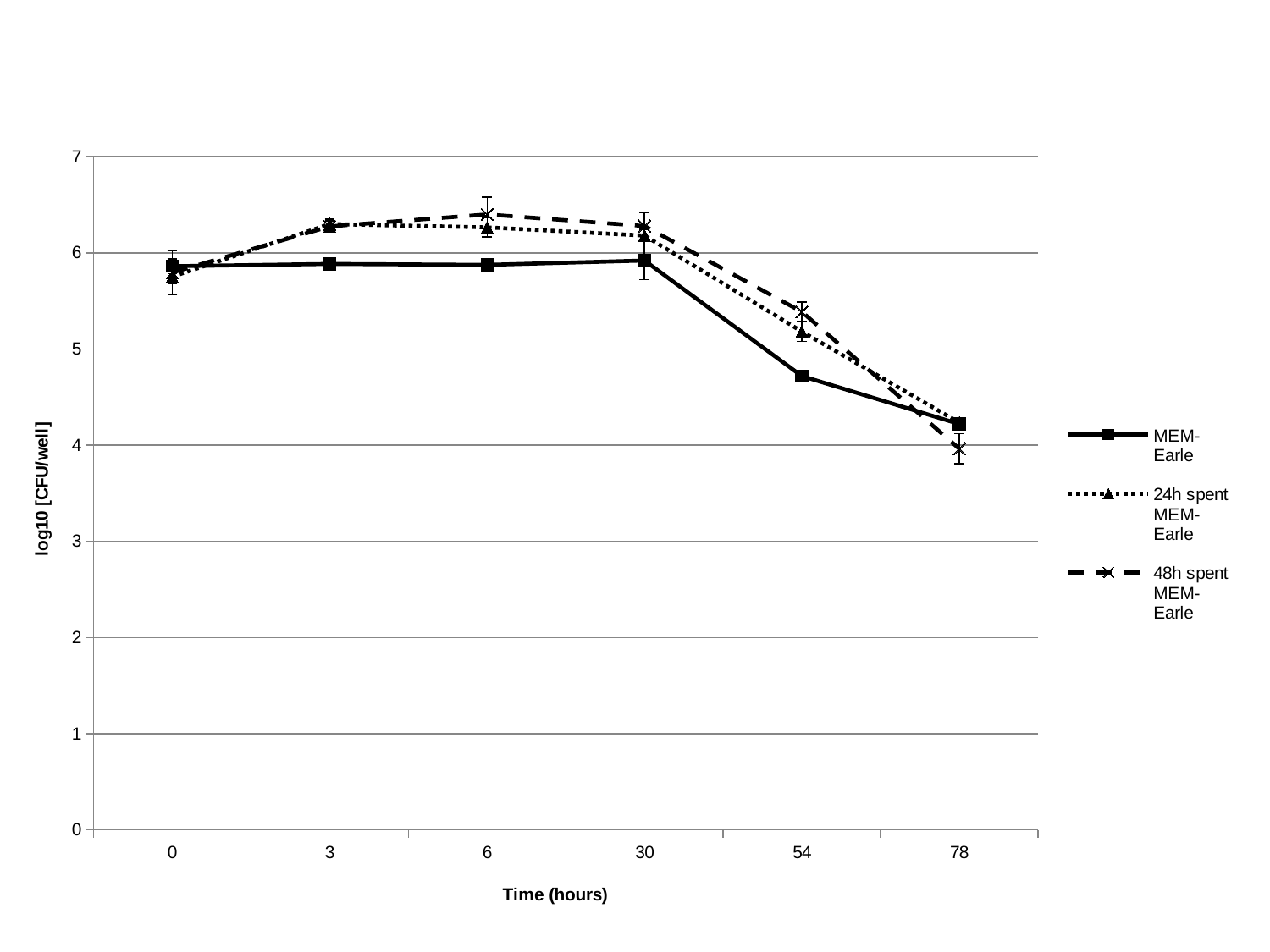

### Chart
| Category | MEM-Earle | 24h spent MEM-Earle | 48h spent MEM-Earle |
|---|---|---|---|
| 0 | 5.86053763630648 | 5.7515228770190685 | 5.794662287175811 |
| 3 | 5.884795363948981 | 6.299216654900513 | 6.274927193099776 |
| 6 | 5.874578445685604 | 6.265211027637486 | 6.399385249546219 |
| 30 | 5.920123326290724 | 6.179934598137441 | 6.278753600952829 |
| 54 | 4.718778397689571 | 5.18089014193745 | 5.386201605400793 |
| 78 | 4.219671830362082 | 4.234685974321529 | 3.9630003484681415 |

Supplement: Additional file 2: — Growth curve of strain JF4278 in fresh and spent MEM-Earle medium. Growth curve showing the survival of M. bovis in fresh and spent medium used for eukaryotic cell culture. [file 13567_2015_194_MOESM2_ESM.pptx]
